# Severe respiratory insufficiency during pandemic H1N1 infection: prognostic value and therapeutic potential of pulmonary surfactant protein A

**DOI:** 10.1186/s13054-014-0479-z

**Published:** 2014-08-07

**Authors:** Monica Fern Tolosa, Nades Palaniyar

**Affiliations:** Program in Physiology and Experimental Medicine, Peter Gilgan Centre for Research and Learning, The Hospital for Sick Children, 686 Bay Street, Toronto, ON M5G 0A4 Canada; Department of Laboratory Medicine and Pathobiology, Faculty of Medicine, University of Toronto, 1 King’s College Circle, Toronto, ON M5S 1A8 Canada; Institute of Medical Sciences, Faculty of Medicine, University of Toronto, 1 King’s College Circle, Toronto, ON M5S 1A8 Canada

## Abstract

For almost two decades, studies have shown collectins to be critical for effective antimicrobial defense of the airways. Members of this protein family, which includes surfactant proteins (SP)-A and D, provide broad-spectrum protection through promoting the aggregation and clearance of pathogens. Interestingly, these proteins may also modulate the immune response, and growing evidence has shown collectins to be protective against several markers of inflammation and injury. In a recent study by Herrera-Ramos and colleagues, genetic variants of collectins were examined in Spanish patients with the pandemic 2009 H1N1 influenza A virus. Comparing genotypes for measures of poor lung function, inflammation, and admission to intensive care, these authors identified three variants of the SP-A gene *SFTPA2* that positively correlated with flu severity. Remarkably, they also found the haplotype 1A^1^ of *SFTPA2* to be protective against these indicators, suggesting that targeted therapy with a recombinant form of SP-A2 may improve patient outcome. Although further work is required to confirm the specificity and efficacy of SP-A in therapeutic H1N1 protection, this study is one of the first to suggest a clinical role for SP-A in pandemic influenza.

## Commentary

Antigenic diversity among circulating influenza A virus (IAV) strains poses a challenge for the adaptive immune system in the rapid control of infection, especially without prior vaccination. However, the innate immune system is equipped to control early stages of viral replication with a repertoire of broad-spectrum, pathogen-recognition molecules called collectins.

In the previous issue of *Critical Care*, Herrera-Ramos and colleagues [1] examined a panel of collectin genes - mannose-binding lectin (*MBL2*), surfactant proteins A1 and A2 (*SFTPA1*/*SFTPA2*), and surfactant protein D (*SFTPD*) - in Spanish patients with pandemic 2009 H1N1 infection. The objective of their study was to identify genetic variants that predisposed patients to severe influenza. The authors defined infection severity by a number of indicators, including poor lung function (requirement for mechanical ventilation, acute respiratory distress syndrome, or acute respiratory failure), hypoxemia (ratio of partial pressure of oxygen in arterial blood to the fraction of inspired oxygen, or PaO_2_/FiO_2_), inflammation (bacteremia or secondary pneumonia), and patient instability (need for intensive care). They identified three polymorphisms (alleles rs1965708-C, rs1059046-A, and the haplotype 1A^0^) that significantly correlated with poor clinical outcome, all of them remarkably for *SFTPA2*.

The indication that *SFTPA2* variants have the greatest effect on IAV control aligns well with previous work on collectin-virus interactions. A majority of the IAV surface is composed of the protein hemagglutinin, which can be modified by glycosylation and made susceptible to collectin binding. However, while surfactant protein (SP)-D and MBL recognize highly glycosylated virions, they have poor efficacy against strains with minor glycosylation, such as pandemic H1N1 [2,3]. In contrast, SP-A acts as a decoy receptor. Sialic acid residues present on the globular domain of SP-A are recognized by hemagglutinin. Therefore, SP-A efficiently neutralizes even the poorly glycosylated strains of influenza [4,5]. Finally, *SFTPA2* expression has been found in both the upper and lower airways, making it a good candidate for H1N1 inhibition in both regions [6, 7].

Overall, the data set presented provides good clinical support for the role of *SFTPA2* in the control of pandemic H1N1, although the mechanism by which SP-A may act is still ambiguous. In theory, SP-A could improve patient outcome through direct viral neutralization or indirectly through maintaining lung homeostasis and constraining the collateral effects of inflammation [8]. Additional measures of viral replication in patient airways may help to clarify whether SP-A is acting in a truly anti-viral or anti-inflammatory manner.

Perhaps it is worth mentioning that the study provided no information about the status of the protein product between patients. In addition to sequence variation, differences in the quantity of SP-A and other collectins may influence clinical outcome [9]. Indeed, other groups have started to uncover subtleties between *SFTPA* alleles and protein translation efficiency *in vitro* [10, 11]. Post-translational regulation and cleavage of SP-A in inflamed lungs may also abrogate the ability of collectin to bind targets [12]. It is possible that some of the genetic variants identified by the current group are subject to different rates of protein turnover in the lungs, with downstream effects on flu severity. However, this rather complex question will require further protein-based examination of patient airways.

Nevertheless, the work presented by Herrera-Ramos and colleagues has identified promising targets for investigation. The variants rs1965708-C (changes amino acid 223 in the globular domain; Q223K) and rs1059046-A (changes amino acid 9 in the signal peptide; T9N) and the haplotype 1A^0^ (differs from A^1^ at both amino acids 9 and 223) may have prognostic value for severe flu, although it is appropriate to note that this study included only patients of Spanish descent. Testing the predictive power of these variants in a broader cohort will be necessary before adopting them as clinical markers.

Finally, the authors identified the *SFTPA2* haplotype 1A^1^ to be protective for a number of indicators (acute respiratory failure, acute respiratory distress syndrome, PaO_2_/FiO_2_ ratio, and mechanical ventilation), suggesting that this variant may be examined for future therapy against pandemic H1N1. Although the idea of delivering a single recombinant protein to improve flu severity may appeal to many of us, we must be mindful that IAV has incredible potential to acquire genetic mutations randomly and under intense selective pressure. Nevertheless, since SP-A is posited to bind IAV through a conserved sialic acid-hemagglutinin interaction, it may have clinical value. Most importantly, if SP-A can also aggressively regulate the host response irrespective of pathogen specificity we may have a potential therapy on our hands.

## Conclusions

Herrera-Ramos and colleagues have demonstrated the value of tailoring treatment for both the patient genotype and influenza strain in exceptional cases of H1N1 infection. They have identified three genetic variants of *SFTPA2* that associate with flu severity (rs1965708-C, rs1059046-A, and haplotype 1A^0^) and one with protection (1A^1^). Continuing efforts to bridge our understanding of genetic variation and flu progression will be a valuable direction for future clinical care.

## Abbreviations

IAV: Influenza A virus
MBL: Mannose-binding lectin
PaO_2_/FiO_2_: Partial pressure of arterial oxygen/fraction of inspired oxygen
*SFTPA*: Surfactant protein A (gene)
*SFTPD*: Surfactant protein D (gene)
SP: Surfactant protein

## References

[1] Herrera-Ramos E, López-Rodríguez M, Ruíz-Hernández JJ, Horcajada JP, Borderías L, Lerma E, Blanquer J, Pérez-González MC, García-Laorden MI, Florido Y, Mas-Bosch V, Montero M, Ferrer JM, Sorlí L, Vilaplana C, Rajas O, Briones M, Aspa J, López-Granados E, Solé-Violán J, Rodríguez De Castro F, Rodríguez-Gallego C (2014). Surfactant protein A genetic variants associate with severe respiratory insufficiency in pandemic influenza A virus infection. Crit Care.

[2] Hartshorn KL, Crouch EC, White MR, Eggleton P, Tauber AI, Chang D, Sastry K (1994). Evidence for a protective role of pulmonary surfactant protein D (SP-D) against influenza A viruses. J Clin Invest.

[3] Job ER, Deng YM, Tate MD, Bottazzi B, Crouch EC, Dean MM, Mantovani A, Brooks AG, Reading PC (2010). Pandemic H1N1 influenza A viruses are resistant to the antiviral activities of innate immune proteins of the collectin and pentraxin superfamilies. J Immunol.

[4] Hartshorn KL, White MR, Shepherd V, Reid K, Jensenius JC, Crouch EC (1997). Mechanisms of anti-influenza activity of surfactant proteins A and D: comparison with serum collectins. Am J Physiol.

[5] Benne CA, Kraaijeveld CA, van Strijp JA, Brouwer E, Harmsen M, Verhoef J, van Golde LM, van Iwaarden JF (1995). Interactions of surfactant protein A with influenza A viruses: binding and neutralization. J Infect Dis.

[6] Madsen J, Tornoe I, Nielsen O, Koch C, Steinhilber W, Holmskov U (2003). Expression and localization of lung surfactant protein A in human tissues. Am J Respir Cell Mol Biol.

[7] Guarner J, Falcón-Escobedo R (2009). Comparison of the pathology caused by H1N1, H5N1, and H3N2 influenza viruses. Arch Med Res.

[8] Palaniyar N (2010). Antibody equivalent molecules of the innate immune system: parallels between innate and adaptive immune proteins. Innate Immun.

[9] Phelps DS, Rose RM (1991). Increased recovery of surfactant protein A in AIDS-related pneumonia. Am Rev Respir Dis.

[10] Silveyra P, Wang G, Floros J (2010). Human SP-A1 (SFTPA1) variant-specific 3’ UTRs and poly(A) tail differentially affect the in vitro translation of a reporter gene. AJP Lung Cell Mol Physiol.

[11] Silveyra P, Raval M, Simmons B, Diangelo S, Wang G, Floros J (2011). The untranslated exon B of human surfactant protein A2 mRNAs is an enhancer for transcription and translation. Am J Physiol Lung Cell Mol Physiol.

[12] Lecaille F, Naudin C, Sage J, Joulin-Giet A, Courty A, Andrault PM, Veldhuizen RA, Possmayer F, Lalmanach G (2013). Specific cleavage of the lung surfactant protein A by human cathepsin S may impair its antibacterial properties. Int J Biochem Cell Biol.

